# The Therapeutic Effect of Second Near-Infrared Absorbing Gold Nanorods on Metastatic Lymph Nodes via Lymphatic Delivery System

**DOI:** 10.3390/pharmaceutics13091359

**Published:** 2021-08-28

**Authors:** Adewale O. Oladipo, Thabang C. Lebepe, Vuyelwa Ncapayi, Ncediwe Tsolekile, Sundararajan Parani, Sandile P. Songca, Shiro Mori, Tetsuya Kodama, Oluwatobi S. Oluwafemi

**Affiliations:** 1 Department of Chemical Sciences, University of Johannesburg Doornfontein Campus, Johannesburg 2028, South Africa; 201504949@student.uj.ac.za (A.O.O.); tlebepe@uj.ac.za (T.C.L.); 216087577@student.uj.ac.za (V.N.); 201504952@student.uj.ac.za (N.T.); PSundararajan@uj.ac.za (S.P.); 2 Centre for Nanomaterials Science Research, University of Johannesburg, Johannesburg 2028, South Africa; 3 Department of Chemistry, University of KwaZulu-Natal, Private Bag X 54001, Durban 4000, South Africa; songcaS@ukzn.ac.za; 4 Graduate School of Biomedical Engineering, Tohoku University, 4-1 Seiryo, Aoba, Sendai 980-8575, Japan; Shiro.mori.a5@tohoku.ac.jp; 5 Department of Oral and Maxillofacial Surgery, Tohoku University Hospital, 1-1 Seiryo, Aoba, Sendai 980-8575, Japan

**Keywords:** lymphatic route, gold nanorods, photothermal therapy, metastatic lymph node, near-infrared light

## Abstract

Photothermal therapy has been established recently as a non-invasive treatment protocol for cancer metastatic lymph nodes. Although this treatment approach shows efficient tumour ablation towards lymph node metastasis, the monitoring and reporting of treatment progress using the lymphatic delivery channel still need to be explored. Herein, we investigated the anti-tumour effect of pegylated gold nanorods with a high aspect ratio (PAuNRs) delivered via the lymphatic route in a mouse model. In this study, breast carcinoma (FM3A-Luc) cells were inoculated in the subiliac lymph node (SiLN) to induce metastasis in the proper axillary lymph node (PALN). The treatment was initiated by injecting the PAuNRs into the accessory axillary lymph node (AALN) after tumour metastasis was confirmed in the PALN followed by external NIR laser irradiation under a temperature-controlled cooling system. The anti-tumour impact of the treatment was evaluated using an in vivo bioluminescence imaging system (IVIS). The results showed a time-dependent reduction in tumour activity with significant treatment response. Tumour growth was inhibited in all mice treated with PAuNRs under laser irradiation; results were statistically significant (** *p* < 0.01) even after treatment was concluded on day 3. We believe that this non-invasive technique would provide more information on the dynamics of tumour therapy using the lymphatically administered route in preclinical studies.

## 1. Introduction

Cancer remains one of the leading causes of mortality worldwide, and most cases are due to lymph node (LN) metastasis [1]. To date, many measures have been developed to combat this trend, with remarkable therapeutic outcomes [2,3,4,5]; however, there is still a growing number of physical and emotional side effects associated with these protocols [6,7]. The destruction of tumour metastasis under hyperthermia has been investigated in the past few years as a non-invasive treatment method [8,9]. For deep-embedded metastatic LN photothermal therapy, the use of gold nanorods with a large cross-section absorption in the second biological NIR window (900–1100) is required [3,10,11,12]. Gold-nanorods’ (AuNRs) unique shape-dependent longitudinal surface plasmon resonance (LSPR) and their optical properties make them an ideal nanoparticle for the laser-induced destruction of tumour cells [13,14,15]. In addition, their ease of functionalisation with targeting ligands for maximum accumulation in the tumour matrix [16,17], biocompatibility for a long-term in vivo existence [18,19,20], and decreased reticuloendothelial clearance [21] make them ideal hyperthermia candidates. Under laser light irradiation, AuNRs absorb light, which is converted into heat, resulting in hyperthermia-induced cell death and subsequent tumour regression [9,22,23,24]. The delivery of anti-cancer agents to metastatic sites is usually achieved through intravenous administration [25,26]. However, only a fraction of the administered drug dosage can reach the target site due to the preferential reabsorption of small anti-cancer drug molecules into blood capillaries [5,27,28]. This often results in insufficient dosage accumulation and retention of the anti-cancer agents required to treat metastasis effectively. In addressing this issue, our group reported a more efficient method for anti-cancer drug delivery to metastatic sites via the lymphatic route [24,29,30]. This delivery route has motivated the design of new nanocomposite-based therapeutic approaches as alternatives to invasive treatment protocols [31,32]. While this delivery route resulted in sufficient dosage delivery to target sites in high concentrations and with effective tumour cell destruction and growth termination under external NIR laser irradiation, little is known about the therapeutic dynamics and treatment prediction.

In the present study, the triangular network of lymph nodes linked together by the lymphatic vessels was used to evaluate and quantitatively monitor the therapeutic opportunities of lymphatically delivered, high-aspect-ratio AuNRs for photothermal applications in a mouse model [33]. NIR laser irradiation at the metastatic lymph node site commenced 160 s after lymphatic administration, with sufficient accumulation in the tumour area within a short time. To assess the antiangiogenic treatment effect and tumour growth luciferase expression, an in vivo bioluminescence imaging system (IVIS) was used. Our results indicate that tumour ablation and growth inhibition using this delivery route is a function of treatment time. This procedure can provide extensive information for the precise control of treatment in preclinical trials.

## 2. Materials and Methods

### 2.1. Materials

Hydrogen tetrachloroaurate hydrate (99.9%), NaBH_4_ (99%), silver nitrate (99%), CTAB (≥99%), ascorbic acid (99%), and NaOL (≥99%) were purchased from Sigma-Aldrich (Kempton Park, South Africa). Hydrochloric acid (ACS reagent, 37 wt%) was obtained from Wako (Osaka, Japan). All chemicals were of analytical grade and were used without further purification. All solutions, other than gold and CTAB, were freshly prepared. The glassware used in the experiments was cleaned and washed thoroughly with MilliQ water and dried before use. A Millipore UV-synergy water purification system of 18.2 MΩ cm @ 25 °C resistivity was used for water production.

### 2.2. PEGylated Gold Nanorod Synthesis

AuNRs were synthesised using a seed-mediated technique adapted from Ye et al. [34], with slight modifications. In a typical synthesis procedure, the seed solution was prepared by adding 0.3645 g of CTAB to 10 mL 40 °C warm water. The solution was allowed to cool to room temperature before adding 0.250 mL of HAuCl_4_ xH_2_O solution (0.01 M) followed by stirring the mixture for 30 min. A freshly prepared solution of 0.6 mL of 0.01 M NaBH4 was added to this solution under vigorous stirring for 2 min to produce a light brown solution that served as the seed solution. The solution was kept at 30 °C for 30 min before use. In a 100 mL Erlenmeyer flask, 0.037 M of CTAB and 0.1234 g NaOL were dissolved in 25 mL of warm water (~40 °C) to prepare the growth solution. The solution was allowed to cool down to 30 °C, and 1.2 mL of AgNO_3_ (4 mM) solution was added, followed by gentle stirring for 15 min. To this solution, 1 mM HAuCl_4_·H_3_O solution was added, and the solution was further stirred for 1 h, followed by pH adjustment by adding 0.96 mL of 37% HCl. After stirring for 15 min, 1.25 mL of ascorbic acid (0.064 M) was added, and the solution was vigorously stirred for 30 s. After this, 4 µL of seed solution was added to the growth solution, and the resultant mixture was stirred for 30 s and left undisturbed at 30 °C for 12 h. The AuNR solution was centrifuged at 7000 rpm for 30 min to remove excess unreacted reagents. AuNR ligand exchange was achieved by mixing 500 µL of AuNRs with 500 µL of 1 mM mPEG-SH (MW 5000) and 100 µL 1X–PBS under magnetic stirring for 12 h. The final mPEG-SH functionalised AuNRs (PAuNRs) were centrifuged to remove any unbound mPEG-SH and sonicated for 10 min. The nanorods were redispersed in 1X–PBS solution for further use.

### 2.3. Characterisation of AuNRs

Absorption spectra were primarily obtained using a JASCO V-770 NIR spectrophotometer. TEM was performed using a JEOL JEM-2100F HRTEM with accelerating voltage of 200 kV. Surface charge measurements were performed using a Photal ELS-Z2MH instrument. Au content in tissues was determined using a PerkinElmer Optima 8300 ICP-OES Spectrometer (Perkin-Elmer, Waltham, MA, USA).

### 2.4. Mice

The mice used in these experiments (2013BeA-019 & 2016BeA-004) were MXH10/Mo-*lpr*/*lpr* [33], bred under specific pathogen-free conditions at the Animal Research Institute, Graduate School of Biomedical Engineering, Tohoku University, Sendai, Miyagi, Japan. They had unique LNs, growing up to 10 mm in size, equivalent to humans at 2.5–3 months of age. In addition, these mice do not develop severe autoimmune diseases.

### 2.5. FM3A-Luc Cell Culture

RPMI-1640 medium (Sigma-Aldrich) (supplemented with 10% heat-inactivated foetal bovine serum, 0.5% Geneticin G418, and 1% L-glutamine–penicillin–streptomycin (Sigma-Aldrich)) was used to cultivate FM3A-Luc (mouse mammary carcinoma cells containing luciferase gene expression) cells, in an incubator under the conditions of 37 °C, 5% CO_2_, and 95% air to achieve 80% confluence [24]. The cells were evaluated using a MycoAlert *Mycoplasma* Detection Kit (Lonza Rockland, Inc, Rockland, ME, USA) before use for the absence of *Mycoplasma* contamination, following the manufacturer’s protocol. 

### 2.6. Metastasis Induction of PALN with Tumour Cells from the SiLN

Metastasis was induced in PALN by injecting a suspension of 3.3 × 10^5^ cells in 20 µL PBS and 40 µL of 400 mg/mL Matrigel (Collaborative Biomedical Products, Bedford, MA, USA) into the unilateral SiLN (*n* = 24) [35]. Briefly, a 27G needle was used to carry out the intranodal inoculation into the SiLN with the assistance of a high-frequency ultrasound imaging system (VEVO770; VisualSonics Fuji Film, Tokyo, Japan) with a 25 MHz transducer (RMV-710B; axial resolution, 70 µm; focal length, 15 mm; VisualSonics FujiFilm). Matrigel solidification after removing the needle was achieved by maintaining the needle in the same position for one minute. The inoculation day was marked as day 0. The metastasis to the PALN was evaluated every three days post-inoculation using an in vivo bioluminescence imaging system (IVIS Lumina; PerkinElmer, Waltham, MA, USA), with a background luciferase activity of ~4 × 10^4^ photons/s. Metastatic mice were considered when the luciferase activity in the PALN was greater than ~5 × 10^5^ photons/s. Luciferin (150 mg/kg, Promega Co., Madison, WI, USA) was injected intraperitoneally to measure luciferase bioluminescence after 10 min for 1 min, using IVIS. The metastatic mice were divided into four groups: Control group (group I), PAuNRs group (group II), Laser group (group III), and PAuNRs + Laser group (group IV). Each group contained five mice. The high-frequency ultrasound system (VEVO770; VisualSonics) was used to measure the PALN volume changes on day 0 and day 18.

### 2.7. In Vivo Evaluation of the Metastatic PALNs Treated with PAuNRs and Laser

In vivo photothermal treatment of PALNs was performed using laser irradiation after injection with PAuNRs. In short, the metastatic group IV mice were intranodally injected with PAuNRs (40 µg/mL, 120 µL) into the AALNs. After 160 s post-injection, the temperature control system (cooling system water temperature, 10 °C; water flow rate, 760 mL/s) was used to cool down the skin temperature to <15 °C before irradiation. The mice PALNs were irradiated with a continuous-wave Nd:YVO_4_ air-cooled NIR laser (2.5 ± 0.5 W/cm^2^; wavelength, 1064 nm; TEM_00_ beam diameter, 0.6 mm; CYD-010-TUBC, Neoarc) with a laser focus diameter of up to 0.6 cm. The PALN’s temperature was maintained at 45 °C for 5 min by tuning the intensity of the laser beam. A K-type thermocouple (Ishikawa Trading Co. Ltd., Tokyo, Japan) was used to measure the site temperature during irradiation. The PAuNRs’ anti-tumour efficacies were evaluated by measuring the luciferase activities in the metastasised PALN on day 0, 1, 2, 3, 4, and 6 after treatment, using IVIS Lumina. The first day of treatment was marked as day zero. Aluminium foil and black cardboard were used to obscure the SiLNs in order to monitor the luciferase activity in the PALNs effectively. The luciferase intensity covered by the region of interest (ROI) around the PALNs was measured. 

### 2.8. PAuNR Biodistribution 

The PAuNRs’ biodistribution was evaluated using inductively coupled plasma optical emission spectroscopy (ICP-OES) by measuring the metal concentration in the mice’s harvested organs. Briefly, after day 6 post-treatment, the mice were sacrificed and harvested. The abdominal aorta blood was drawn, and the organs and tissues were collected and freeze-dried for 20 h. Freeze-dried samples were digested in aqua regia and analysed using ICP-OES for metal concentration. 

### 2.9. Histological Analysis

To prepare samples for histological analysis, the harvested organs were fixed in 10% formaldehyde in PBS (Rapid Fixative, Kojima Chemical Industry, Saitama, Japan) for 4 days at 4 °C, followed by dehydration, and, lastly, they were embedded in paraffin. The paraffin-embedded organs were sliced into 2–4 µm sections. Immunohistochemistry (IHC) staining for anti-CD 31 was carried out using an automated staining processor (Discovery XT, Ventana Medical Systems, Inc., Oro Valley, AZ, USA). The vascular endothelial cells’ IHC staining was carried out in pre-diluted polyclonal goat anti-CD31 antibody (1/100 dilution; sc-1506, Santa Cruz Biotechnology, Inc., Dallas, TX, USA; 2 h at room temperature) combined with a biotinylated anti-goat IgG (H + L) (BA5000, Vector Laboratories, Burlingame, CA, USA; 20 min at room temperature). Polyclonal rabbit anti-LYVE-1 antibody diluted in 1:250 (103-PA50AG, ReliaTech GmbH, Wolfenbüttel, Germany; 2 h at room temperature) combined with a biotinylated anti-rabbit IgG (H + L) (BA1000, Vector Laboratories, Burlingame, CA, USA; 20 min at room temperature) was used to carry out the IHC staining of vascular endothelial cells. A BX-51 Olympus microscope connected to a digital camera (DP72; Olympus corporation, Tokyo, Japan) was used to determine the specimen boundary at low magnification.

### 2.10. Statistical Analysis

All measurements are presented as the mean ± SEM. Differences between any two groups were determined by one-way ANOVA followed by the Mann–Whitney test. A *p* value < 0.05 was considered to represent a minimal statistically significant level. Box and whisker plots displaying the first, second (median), and third quartiles were used to represent the data. Both ends of the whiskers represent the maximum and minimum values of all data.

## 3. Results

### 3.1. Synthesis and Characterisation of PAuNRs

**Figure 1:**
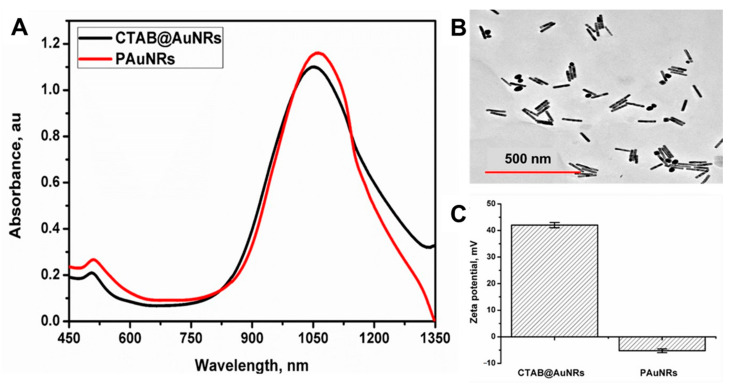
(**A**) Absorption spectra of AuNRs and PAuNRs. (**B**) TEM image of AuNRs (scale bar = 500 nm). (**C**) Surface charge (zeta-potential) of AuNRs and PAuNRs (mean ± SD, *n* = 3).

AuNRs with an aspect ratio of 6.71 were fabricated according to a previously reported protocol (see Materials and Methods) [34]. Many reports on the toxicity of AuNRs have been presented due to hexadecyltrimethylammonium bromide (CTAB), the surfactant used for the fabrication [11,18,36]. Therefore, to improve the biocompatibility of AuNRs, ligand exchange of CTAB with polyethylene glycol (PEG) was employed. The PAuNRs showed a slight red shift in LSPR by 3 nm after ligand exchange (Figure 1A). The TEM image of PAuNRs in Figure 1B shows rod-shaped NPs with an average length of 92.9 nm and width of 6.1 nm. The zeta-potential of AuNRs after ligand exchange with PEG changed from +42.01 mV to −5.27 mV (Figure 1C). This change in the surface charge is attributed to the successful ligand exchange of the CTAB-capped AuNRs with the PEG molecule coating. The obtained PAuNRs’ size and charge make them an ideal material for delivery via a lymphatic system because they can be efficiently absorbed into the lymphatic vessels [37]. 

### 3.2. In Vivo Time-Mediated Anti-Tumour Therapy Evaluation

**Figure 2:**
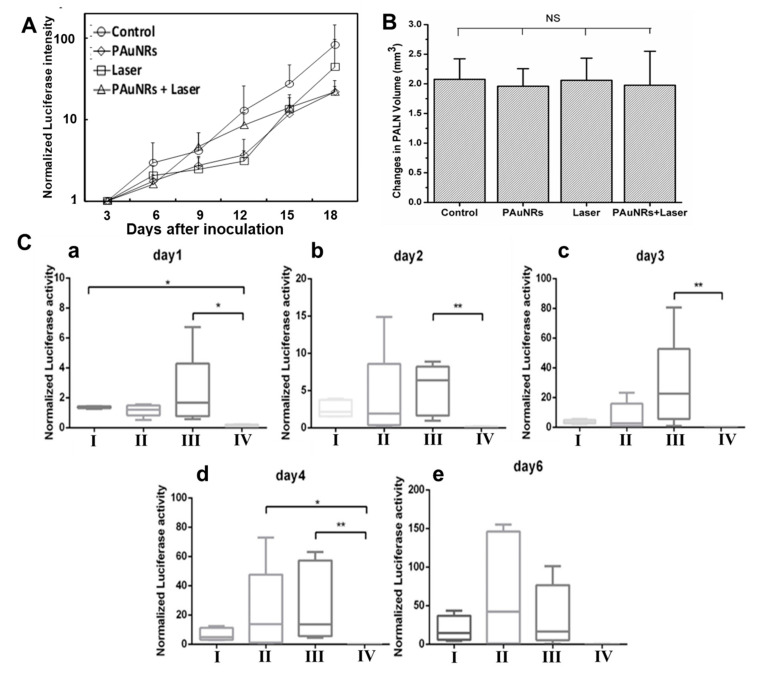
Confirmation of metastasis in the PALN (*n* = 6). (**A**) Normalised luciferase intensity in PALN measured after tumour cell inoculation, and (**B**) PALN volume changes measured before tumour cell inoculation and after metastasis was confirmed. (**C**) Illustration of normalised luciferase intensity in PALN measured after treatment (*n* = 6). (**a**) day 1, (**b**) day 2, (**c**) day 3, (**d**) day 4, (**e**) day 6. NS (Not significant), * *p* < 0.05, ** *p* < 0.01. Error bars represent mean ± SEM.

PAuNRs have been reported to accumulate efficiently in solid tumours due to passive targeting attributed to the EPR effect. The PEG polymer offers increased circulation time in the bloodstream (half-life 17 h) and improved biodistribution using mice models [38,39]. We anticipate that enhanced circulation and accumulation in the metastatic environment can be obtained using the lymphatic delivery route. Since the PAuNRs’ accumulation and retention in the metastatic LN can be enhanced by using the lymphatic delivery route, we proceeded to evaluate and monitor their in vivo anti-tumour activity as a function of time. Prototype mice were created using MXH10/Mo/lpr mice with unique peripheral LNs growing up to 10 mm in size, which is equivalent to humans. In 24 mice, FM3A/Luc cells were injected intranodally into the mice’s SiLN to induce tumour metastasis in the PALN. Mice were divided into four groups: group I consisted of mice injected with PBS at AALN without laser irradiation as a control group, group II consisted of mice injected with PAuNRs at AALN without laser irradiation, group III consisted of mice injected with PBS at AALN followed by laser irradiation at PALN on day 0, 1, 2, and 3, and the last group of mice were injected with PAuNRs at AALN followed by laser irradiation at PALN on day 0, 1, 2, and 3 (group IV). It is worth noting that the CTAB-capped AuNRs were not used in these experiments because of the toxicity that may arise from the CTAB [24]. IVIS Lumina was used to monitor the luciferase-expressing enzyme found in the cell activity, which enabled us to monitor the tumour growth in the SiLN as well as confirm metastasis in the PALN. The growth progression of the tumours in the SiLN and the formation of metastasis in the PALN were monitored every 3 days (Figure 2A). Metastasis in the downstream PALN of the mice started to develop after 2 weeks post-inoculation, as tumour cells continued to flow into the capsule from SiLN. This was characterised by increased luciferase activity in the PALN of each group after inoculation. Furthermore, 3D high-frequency ultrasound (VEVO-770) was used to measure the changes in the volume of the PALN. There was no significant difference in the volume changes of the PALN of each group by the end of 18 days after inoculation (Figure 2B). These results confirm the establishment of metastasis in the PALN (~5 × 10^5^ photons/s). PAuNRs (120 µL of 40 µg/mL) and phosphate-buffered saline (PBS of 120 µL) were administered to mice of each group on day 0 and day 2 from the AALN after metastasis was confirmed in the PALN.

Laser irradiation of metastatic PALN commenced 160 s after PAuNR injection in the AALN. The metastatic PALN was then irradiated with an NIR laser for 300 s after the LN surface was water-cooled to <15 °C to prevent skin damage. The tumour activities of the mice in all groups were observed daily and then compared with each other. The optical changes related to the luciferase activities of tumour cells presented by in vivo bioluminescence imaging afforded us the opportunity to monitor treatment progress in real time. The variability in the treatment effect of each group was considered for further statistical analysis by normalising the luciferase intensities in the PALNs to the background. The tumours of group IV immediately showed negligible luciferase activity from day 1 when compared with the other groups. Smaller areas of luciferase activity were observed in groups I and group II, indicating similar tumoural activities. For the laser group, which was group III, the luciferase activity increased to the maximum, with 75% of mice showing tumour activity below 5 (Figure 2(Ca)). In group IV, tumour activity was reduced significantly as early as day 1 (* *p* < 0.05). This demonstrates the immediate anti-tumour effect of the combined PAuNRs with the laser. On day 2, fresh injections of PAuNRs and PBS were performed in the groups to start the next round of treatment. Tumour activity in the control, PAuNR, and laser groups increased compared to day 1. Group II had a lower median, indicating that 50% of mice had lower tumour activity, yet 50% showed a 2–15% tumour growth increase. Although the laser group’s (group III) maximum and minimum luciferase intensity were less than those of the PAuNR group (group II), the whiskers suggest similar tumour behaviour, with roughly 50% of mice showing higher tumour activity when compared to day 1. The PAuNRs + laser group (group IV) showed a similar downward trend of reduced tumour activity compared to the other groups. The statistical difference of ** *p* < 0.01 indicates a remarkable improvement, suggesting a further disruption of tumour angiogenesis and proliferation. Here, a ~100% tumour regression result from all mice receiving metastasis treatment in this group was achieved. Figure 2(Cb) shows the result on day 2, which suggests that both group II and III did not display any therapeutic effect. Continuous tumour proliferation was observed in groups I, II, and III on day 3. Among these three groups, group III demonstrated a rapid luciferase increase despite laser irradiation. Around 50% of the mice in this group showed luciferase activity above 20, which was significantly higher than the previous day. A similar increase was also observed in group II. In contrast, group IV continued to demonstrate an efficient therapeutic effect based on its negligible luciferase intensity on day 3 (Figure 2(Cc)). A statistical difference of ** *p* < 0.01 indicated a marked contrast in anti-tumour effect when compared to the other groups. Groups I, II, and III’s tumour luciferase activity continued to increase on day 4 (Figure 2(Cd)). Group II’s luciferase activity showed a rapid increase each day, with the maximum values increasing from 24 on day 3 to 76 on day 4, and 50% of mice having between 15 and 76 luciferase intensity. However, the maximum luciferase intensity value for group III decreased from 80 on day 3 to 60 on day 4, indicating some anti-tumour effect. The median reduced from 22 on day 3 to 14 on day 4 (50% of mice on both days). Nonetheless, this was not significant enough to cause a disruption in tumour cell growth within the metastatic area. The near-negative luciferase intensity further emphasised the role of hyperthermia-induced cell death caused by PAuNRs under laser irradiation in group IV. Compared with the other groups, * *p* values of 0.05 and 0.01 were considered statistically significant differences. After a one-day interval, the therapeutic effects of the protocol were evaluated to determine tumour dynamics after day 6. We discovered aggressive tumour activity in all the groups except group IV (Figure 2(Ce)). The maximum luciferase intensities of the control, PAuNRs, and laser group increased with the lower shifting of the median in the box. However, the medians were higher than the values obtained on day 4, and 75% of all the mice in these groups showed continuous tumour proliferation after treatment. We attribute this occurrence to the fact that tumour activities in the PALN of mice are a function of several factors, including the immune response to the tumour, body conditions, etc. Unlike other groups, negative luciferase intensity characterised the tumour growth activity in group IV, signifying complete tumour destruction long after treatment ended on day 3.

**Figure 3:**
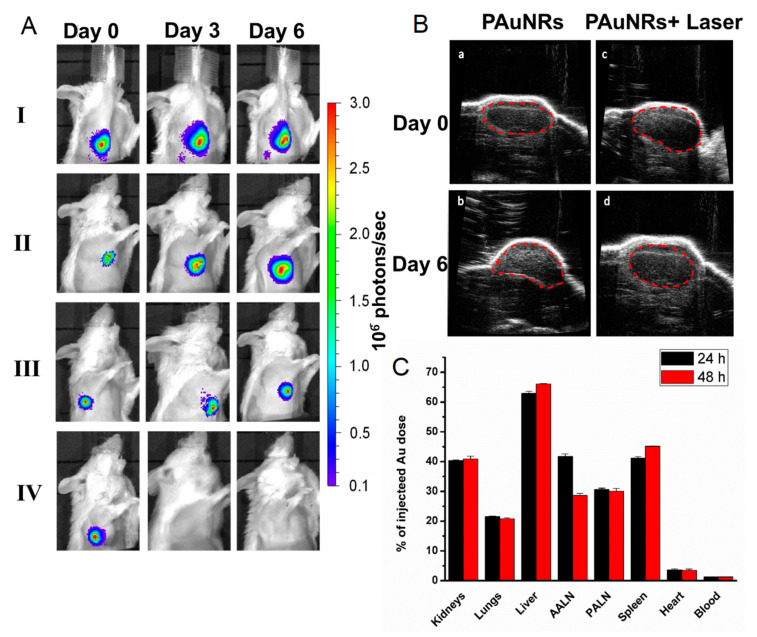
Anti-tumour evaluation of the treatment. (**A**) Representative images of tumour activities in the PALN of mice detected using an in vivo bioluminescence imaging system (IVIS) from day 0 to day 6 after treatment (*n* = 6). (**B**) Temporal changes in the lymph node size of PAuNRs and PAuNRs + laser groups using two-dimensional (2D) high-frequency ultrasound images. Dashed lines indicate PALN regions. (**C**) Biodistribution of PAuNRs in the tissues measured as Au content using ICP-OES (*n* = 3).

Significant metastasis progression within the groups was also monitored using the intensity of light emission from the luciferase enzyme expression of tumour cells via IVIS. Figure 3A shows that in groups I, II, and III, light intensity increased from the PALN region as the treatment time continued. The photon flux density in the red region gradually expanded in size, signifying the intense luciferase activity of infiltrating tumour cells within the cortex of the lymph nodes. As the primary tumour grew, the lymphatic vessels further increased in size, allowing tumour cells to flow into the PALN, causing a partial replacement of lymphocytes within the lymph nodes [14]. This replacement thus led to a noticeable increase in the PALN size, as shown in the ultrasound image in Figure 3B. However, an immediate treatment effect was noticed in group IV after day 0 by the disappearance of luminescence light and was not visible up to the final day of treatment. This result confirms the usefulness of our proposed short-term lymphatic administration and sufficient drug accumulation for the metastatic lymph node treatment.

To assess the biodistribution kinetics of PAuNRs in vivo after lymphatic administration, we analysed the Au elemental content in tissues by inductively coupled plasma optical emission spectrometry (ICP-OES) at 24 h and 48 h post-administration (120 µL, 40 µg/mL). The result shows a significant accumulation of PAuNRs in the PALN at 24 h, which was maintained even after 48 h post-injection (Figure 3C). In addition, the reticuloendothelial system (liver and spleen) retained the highest accumulation at both 24 and 48 h after injection, while a significant concentration reduction was observed in the other organs.

**Figure 4:**
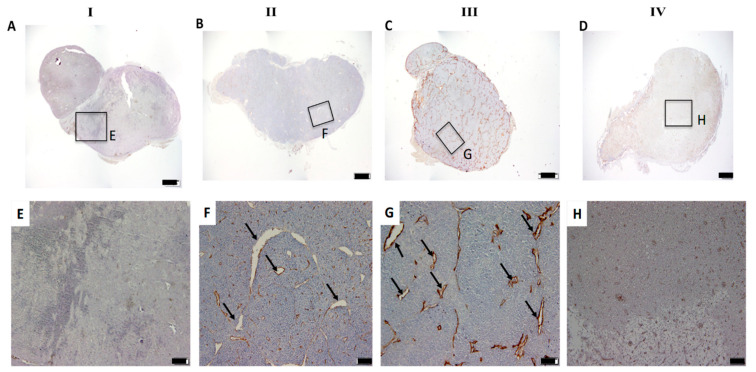
Histological analysis of PALN for anti-tumour effects. Representative section showing immunostaining with anti-CD31 antibody in the whole PALNs of (**A**) control group with PBS only, (**B**) control group with PAuNRs only, (**C**) group with PBS + laser and (**D**) group with PAuNRs + laser after treatment. Scale bars: 200 μm. Magnified view of (**E**) control group with PBS only, (**F**) control group with PAuNRs only, (**G**) group with PBS + laser and (**H**) group with PAuNRs + laser after treatment that is outlined in boxes. Scale bars: 100 μm; I, control group with PBS only; II, control group with PAuNRs only; III, group with PBS + laser; IV, group with PAuNRs + laser; black arrows pointing at the open lumen.

Since tumour growth occurs mainly by angiogenesis (tumours forming their own blood vessels for supply) [40], we further monitored the effectiveness of this treatment protocol by immunohistological analysis. Figure 4 shows the representative images of the treated PALN stained with anti-CD31 antibody, which distinguishes between the tumour blood vessel area and non-tumour area. In the PALN region for group IV, they were much smaller in density compared to the other two groups (II and III groups). The enlarged open lumen seen in group II and III might be caused by the metal nanoparticles and laser when used separately, as they have been shown to cause cell proliferation [24]. This was observed earlier when analysing luciferase intensity using IVIS and can be attributed to the expanded lymphatic vessels caused by rapid tumour growth in the PALN. The observed tumour blood vessel changes in group IV indicate the tumour network vessels’ disruption by combining PAuNRs with laser-induced hyperthermia effects (Figure 4D,H). This inhibited the supply of nutrients to the tumour vasculature and prevented the tumour’s growth capability. This result further confirms the effectiveness of the lymphatically administered PAuNRs in causing thermal damage to tumour cells and inhibiting their proliferation.

Comparatively, there was a significant difference in the therapeutic outcomes between the groups during the treatment period for the control groups (PBS and PAuNRs). The PAuNRs and laser groups showed increased luciferase activities, to a similar degree, at the end of the treatment period. The time course effect of the therapy differed significantly within the groups, with a faster therapeutic effect in the PAuNRs + laser group. In this regard, our results reveal that if the therapeutic effect is due to the first treatment only, the additional treatments administered should not alter the course of treatment evolution with regard to time and response rate. We have also shown that the responsiveness of aggressive metastatic cells within the PALN to therapeutic effects could be due to the frequency of treatment administration.

## 4. Conclusions

In summary, we have reported the therapeutic effect of second near-infrared absorbing gold nanorods on metastatic lymph nodes delivered via a lymphatic delivery system in a mouse model under laser irradiation. The results showed that tumour growth was completely inhibited in all mice treated with PAuNRs under laser irradiation, and the findings were statistically significant (** *p* < 0.01) even after treatment was concluded on day 3. In addition, the metastasis treatment through the lymphatic route was time-dependent, with a significant treatment response. Furthermore, the therapeutic effect could be monitored and quantified accurately using the in vivo bioluminescence imaging system. The frequency of administering the anti-cancer agent or laser irradiation did not result in any improvement in the treatment procedure except for the laser irradiation-induced hyperthermia effect in group IV, indicating the promising potential of this protocol for the preclinical monitoring of treatment outcomes for future therapeutics.

## Data Availability

Data available on request due to restrictions.
